# The Impact of Vitamin D_**3**_ Supplementation on Mechanisms of Cell Calcium Signaling in Chronic Kidney Disease

**DOI:** 10.1155/2015/807673

**Published:** 2015-05-04

**Authors:** Ingrid Lajdova, Viera Spustova, Adrian Oksa, Zuzana Kaderjakova, Dusan Chorvat, Marcela Morvova, Libusa Sikurova, Alzbeta Marcek Chorvatova

**Affiliations:** ^1^Department of Clinical and Experimental Pharmacology, Faculty of Medicine, Slovak Medical University, 833 03 Bratislava, Slovakia; ^2^Department of Nuclear Physics and Biophysics, Faculty of Mathematics, Physics and Informatics, Comenius University, 833 03 Bratislava, Slovakia; ^3^Department of Biophotonics, International Laser Centre, 833 03 Bratislava, Slovakia

## Abstract

Intracellular calcium concentration in peripheral blood mononuclear cells (PBMCs) of patients with chronic kidney disease (CKD) is significantly increased, and the regulatory mechanisms maintaining cellular calcium homeostasis are impaired. The purpose of this study was to examine the effect of vitamin D_3_ on predominant regulatory mechanisms of cell calcium homeostasis. The study involved 16 CKD stages 2-3 patients with vitamin D deficiency treated with cholecalciferol 7000–14000 IU/week for 6 months. The regulatory mechanisms of calcium signaling were studied in PBMCs and red blood cells. After vitamin D_3_ supplementation, serum concentration of 25(OH)D_3_ increased (*P* < 0.001) and [Ca^2+^]_i_ decreased (*P* < 0.001). The differences in [Ca^2+^]_i_ were inversely related to differences in 25(OH)D_3_ concentration (*P* < 0.01). Vitamin D_3_ supplementation decreased the calcium entry through calcium release activated calcium (CRAC) channels and purinergic P2X_7_ channels. The function of P2X_7_ receptors was changed in comparison with their baseline status, and the expression of these receptors was reduced. There was no effect of vitamin D_3_ on P2X_7_ pores and activity of plasma membrane Ca^2+^-ATPases. Vitamin D_3_ supplementation had a beneficial effect on [Ca^2+^]_i_ decreasing calcium entry via CRAC and P2X_7_ channels and reducing P2X_7_ receptors expression.

## 1. Introduction

Vitamin D hormonal system has been classically implicated in the regulation of calcium homeostasis and bone metabolism. However, it has also noncalciotropic effects through the activation of tissue vitamin D receptors (VDR) [1]. Vitamin D insufficiency/deficiency is a significant risk factor for the development of various chronic diseases, and the deficiency of calcidiol (25(OH)D_3_) as well as calcitriol (1,25(OH)_2_D_3_) is common in CKD patients [2]. Therefore, the supplementation of native vitamin D (cholecalciferol or ergocalciferol) or active vitamin D (calcitriol and VDR activators) in CKD is well established.

Free cytosolic calcium concentration ([Ca^2+^]_*i*_) is controlled by mechanisms that regulate Ca^2+^ entry from the extracellular space and Ca^2+^ release from intracellular stores and by the activity of ATP-dependent Ca^2+^ pumps and antiporters that move Ca^2+^ back into stores or out of cells [3]. Already in early stages of chronic kidney disease (CKD), [Ca^2+^]_*i*_ and calcium concentration of intracellular stores were significantly increased in comparison with healthy volunteers, and the regulatory mechanisms of calcium signaling were impaired by the disease [4–7].

Calcium enters into the cells by any of the general classes of calcium/cation channels. In nonexcitable cells like peripheral blood mononuclear cells (PBMCs), the predominant Ca^2+^ entry pathway is the store-operated one, in which the emptying of intracellular Ca^2+^ stores activates the Ca^2+^ influx. This type of the channel is known as the calcium release activated calcium (CRAC) channel in lymphocytes. The calcium entry through CRAC channels activates certain transcription factors which regulate the gene expression for cytokines responsible for immune responses [8, 9].

Another mechanism of calcium entry into the cell is represented by purinergic P2X receptors. At the present time, purinergic signaling is accepted as a crucial component of diseases and was found to mediate a vast array of biological processes. The P2X_7_ receptors are expressed primarily on cells of hemopoietic origin, where they participate in immune responses, cell proliferation, cell death, bone formation, and bone resorption [10]. The P2X_7_ receptor is a bifunctional purinoreceptor that opens a nonselective cation channel and consecutively forms a large, cytolytic pore. The key factor of P2X_7_-dependent cytotoxicity is the massive intracellular Ca^2+^ increase triggered by its activation. This can lead to membrane blebbing and cell death by apoptosis or necrosis. There is an increasing body of evidence implicating P2X_7_ receptors in various pathological conditions [11–14].

The plasma membrane Ca^2+^-ATPases (PMCA) is responsible for removing excessive Ca^2+^ out of the cells to extracellular environment. The decreased PMCA activity increases [Ca^2+^]_*i*_ and affects some intracellular processes.

To our knowledge, little information is available regarding the impact of vitamin D_3_ supplementation on disturbed cell calcium homeostasis in CKD. Therefore, the aim of the present study was to examine the effect of vitamin D_3_ supplementation on essential regulatory mechanisms of cell calcium homeostasis.

## 2. Materials and Methods

### 2.1. Patients

The study population consisted of 16 nondiabetic patients with CKD (9 patients CKD stage 2 and 7 patients CKD stage 3). All of them were screened and followed up in the outpatient department of nephrology at the Slovak Medical University. The diagnosis of CKD was based on clinical and laboratory examinations as defined by the K/DOQI criteria [15]. Causes of their renal disease were glomerulonephritis in 9 patients, tubulointerstitial nephritis in 3 cases, hypertensive nephroangiosclerosis in 2 patients, and other in 2 causes. The glomerular filtration rate was estimated by the MDRD study formula [16]. Patients with acute impairment of renal function, nephrotic proteinuria, malignancies, and derangements in mineral metabolism of nonrenal origin were excluded from the study. Concurrent treatments interfering with mineral metabolism were not allowed. Previous therapy with vitamin D_2_/D_3_, calcitriol, or over-the-counter vitamin D preparations had to be cancelled at least 2 months before enrollment. Hypertension was the most common comorbidity present in all patients and treated with ACE inhibitors or angiotensin II receptor blockers in 14, diuretics in 8, betablockers in 6, and calcium channel blockers in 8 cases. Dihydropyridine calcium channel blockers were allowed as they do not interfere with studied parameters and effects in PBMCs [17]. All patients had vitamin D deficiency (serum 25(OH)D_3_ concentration <30 ng/mL) and were supplemented with cholecalciferol 7000–14000 IU/week for 6 months; the dose (approximately 1000–2000 IU/day) was chosen as a common supplementary dose for the treatment of vitamin D deficiency in general population.

The study was approved by the Ethics Committee of the Slovak Medical University and all participants gave their written informed consent.

### 2.2. PBMCs Isolation

Human PBMCs were isolated by the Ficoll gradient centrifugation, diluted 1 : 1 with RPMI-1640 medium, layered onto an equivalent volume of medium LSM-1077, and centrifuged at 700 g for 20 min at 22°C, as previously reported [5]. The PBMC layer was washed in 40 mL RPMI, resuspended in 10 mL RPMI and 10% fetal bovine serum (FBS), and centrifuged at 300 g 10 min at 22°C and the pellet was resuspended in 2 mL aliquots of physiological salt solution. Final concentration of PBMCs was adjusted to 2.5 × 10^6^ cells/mL. Our preparation contained lymphocytes (94–96%), monocytes (3-4%), and natural killer cells (the rest), as determined by flow cytometry (Coulter Epics XL, Ireland). The cell viability was quantified using a 0.8% solution of trypan blue and estimated to be 96–98%. 

### 2.3. Red Blood Cell Membranes Isolation

Isolated red blood cell (RBC) membranes were used to assess the PMCA function. RBC membranes were obtained by hemolytic fragmentation in hypotonic media using standard method of Hanahan and Ekholm [18] modified in our laboratory to achieve a higher quality of the ghosts. RBCs from previous isolation were diluted 1 : 5 with physiological salt solution and centrifuged at 1270 g for 20 min at 4°C. The supernatant was removed and the procedure was repeated one more time. RBCs were then diluted 1 : 5 with tris(hydroxymethyl)aminomethane (TRIS) medium (20 mmol/L, pH = 7.4) and centrifuged at 7700 g for 35 min at 4°C. This step was repeated twice for each TRIS medium with decreasing concentrations (20, 10, and 5 mmol/L).

### 2.4. Intracellular Ca^2+^ Measurements

The population of 2 × 10^6^ PMBCs/mL was loaded with fluorescence dye Fluo-3 AM at a final concentration of 2 *μ*mol/L for 40 min at 22°C in a physiological salt solution. After incubation, the cells were centrifuged at 300 g, washed three times with a physiological salt solution, and kept at room temperature for 10 min before use. The Fluo-3 fluorescence was measured at 37°C in Fluorolog 3–11 spectrofluorometer (HORIBA Jobin Yvon Inc., Edison, NJ, USA) with an excitation at 488 nm (bandpass 3 nm) and an emission at 526 nm (bandpass 5 nm). Each experiment was followed by [Ca^2+^]_*i*_ calibration to estimate the actual free cytoplasmic calcium concentration from the measured fluorescence signal (*F*) in each cell population. [Ca^2+^]_*i*_ was quantified in nmol/L according to the following equation:

(1) $$\begin{array}{c} {\left[{\text{Ca}}^{2+}\right]}_{i}=K_{d}\cdot \left[\frac{F-F_{\min}}{F_{\max}-F}\right], \end{array}$$

where *K*_*d*_ = 400 nmol/L at 37°C [19]. The maximal fluorescence intensity (*F*_max⁡_) was assessed by the addition of Triton X-100 (0.1%) with Ca^2+^ (5 mmol/L), and the minimum fluorescence level (*F*_min⁡_) was determined after the addition of 25 mmol/L EGTA (pH = 9). Digitonin (20 *μ*mol/L) was used to answer for minimal compartmentalization [20]. To assess the role of CRAC channels in Ca^2+^ entry, 2-aminoethyl-diphenyl borate (2APB), a widely used inhibitor of these channels was applied [21]. Although there are already more potent and selective inhibitors of these channels [22], 2APB was used for the possibility of comparing the results with our previous studies [4, 6]. The action of 2APB (50 *μ*mol/L) was studied in cells where Ca^2+^ entry through these channels was stimulated by thapsigargin (Tg) (1 *μ*mol/L), a specific inhibitor of endoplasmic reticulum Ca^2+^-ATPase. To examine the function of P2X_7_ receptors, AZ11645373 (50 nmol/L), a highly selective antagonist of human P2X_7_ receptors [23], and 2′,3′-*O*-(4-benzoyl)benzoyl ATP (BzATP) (50 *μ*mol/L), the most potent and selective agonist of these receptors, were used [24].

### 2.5. Cell Surface P2X_7_ Receptors Expression

PBMCs were stained with P2X_7_ polyclonal antibody labelled with fluorescein isothiocyanate (FITC) according to the protocol provided by the manufacturer. Briefly, 100 *μ*L of PBMCs were incubated either with FITC-conjugated anti-P2X_7_ (2 *μ*g/mL) or FITC-conjugated IgG2a (2 *μ*g/mL, control) for 20 min at 22°C in the dark. PBMCs were simultaneously stained with phycoerythrin- (PE-) conjugated CD14 antibody in order to exclude monocytes from the examined population. After the incubation, cells were washed three times, dissolved in 500 *μ*L of PBS, and subjected to the analysis on flow cytometer (Cytomics FC 500 cytometer, Beckman Coulter, USA). Values of control samples stained with FITC-conjugated IgG2a were subtracted from the evaluated results.

### 2.6. P2X_7_ Receptors Visualization by Fluorescence Microscopy

To visualize the surface P2X_7_ receptors, PBMCs were stained with P2X_7_ (extracellular) antibody. Cell imaging was performed by using the Axiovert 200 inverted microscope (Carl Zeiss, Germany) with mercury lamp HBO-100 and FluoArc driver. The 450–490 nm bandpass filter and FT510 dichroic mirror were used for excitation, and emission was detected with a 515 nm long-pass filter. Images were recorded by the PentaMax cooled CCD camera (Roper Scientific) with KODAK KAF-1400 chip. Images were taken with the C-Apochromat 40x/1.2 water immersion objective (Zeiss, Germany).

### 2.7. Ethidium Bromide Uptake by Flow Cytometry

The ethidium bromide uptake by PBMCs was measured on the Cytomics FC 500 cytometer (Beckman Coulter, USA) and results were processed with the CXP software. PBMCs at concentration of 10^6^ cells/100 *μ*L were kept at room temperature when ethidium bromide at 30 *μ*mol/L final concentration was added to the sample. After a 5 min incubation, the mean channel ethidium fluorescence was assessed in FL3 sensor after excitation with a 488 nm laser beam. Up to 10000 events were involved in the data analysis. For the investigation of P2X_7_ pore function, ethidium fluorescence was measured after a 10 min incubation with either BzATP (50 *μ*mol/L) or AZ11645373 (50 nmol/L) at 37°C.

### 2.8. PMCA Activity Measurement

The RBCs membrane suspension was added to working medium (mmol/L): 100 TRIS, 80 KCl, 3 MgCl_2_, 0.2 ethylenediaminetetraacetic acid (EDTA), and 1 ouabain (pH = 7.4) in the presence or absence of CaCl_2_ (5 mmol/L). The reaction was started by the addition of 40 mmol/L of ATP, conducted for 60 min at 37°C and stopped by the addition of a 15% trichloroacetic acid (TCA). The amount of liberated inorganic phosphate was determined using a phosphate colorimetric assay kit (BioVision) on the UV–VIS spectrophotometer Shimadzu UV-1700 (Shimadzu Corp., Japan). The estimated PMCA activity was calculated as the difference between the activities of the enzyme incubated in the presence and absence of CaCl_2_ and was expressed as nmol of Pi/mg protein/h. The protein concentration was measured by the method of Lowry [25].

### 2.9. Reagents

The physiological salt solution contained (mmol/L) 140 NaCl, 5.4 KCl, 1 CaCl_2_, 1 Na_2_HPO_4_, 0.5 MgCl_2_, 5 glucose, and 5 HEPES (pH = 7.4). Thapsigargin (Tg) was procured from Calbiochem (San Diego, CA). 2-Aminoethyldiphenyl borate (2APB), ethidium bromide, AZ11645373, and 2′,3′-*O*-(4-benzoyl) benzoyl ATP (BzATP) were obtained from Sigma-Aldrich (St. Louis, MO, USA). Fluo-3 acetoxymethylester (Fluo-3 AM) was from Molecular Probes (Eugene, OR), lymphocyte separation medium LSM-1077 was from PAA Laboratories GmbH (Pasching, Austria), and fetal bovine serum (FBS) and RPMI-1640 medium were from GIBCO (Grand Island, NY, USA). PE-conjugated anti-CD14, FITC-conjugated IgG2a, and anti-P2X_7_ (extracellular) FITC were from Sigma-Aldrich (St. Louis, MO, USA). The phosphate colorimetric assay kit was from BioVision (Hayward, CA, USA). All other chemicals were purchased from Sigma-Aldrich.

### 2.10. Analytical Procedures

Serum calcium and creatinine were measured by the Vitros 250 Analyzer, Johnson & Johnson, Rochester, NY, USA. Intact parathormone and 25-hydroxyvitamin D_3_ were determined by the electrochemiluminescence immunoassays (ECLIA) (Roche-Diagnostics, Mannheim, Germany).

### 2.11. Statistical Analyses

All values are expressed as means ± SD. Statistical analysis was carried out by the SPSS 15.0 (SPSS Inc., Chicago, IL, USA). The Shapiro-Wilk test was used to evaluate a sample normality distribution. The statistical significance of differences was tested by the independent 2-population Student's* t-*test for normally distributed data and the Wilcoxon's test for a nonparametric analysis. A *P* value < 0.05 was considered significant. The Spearman's correlations between variables were used as a measure of association. 

## 3. Results

### 3.1. Effect of Cholecalciferol Treatment on Main Laboratory Variables

**Figure 1:**
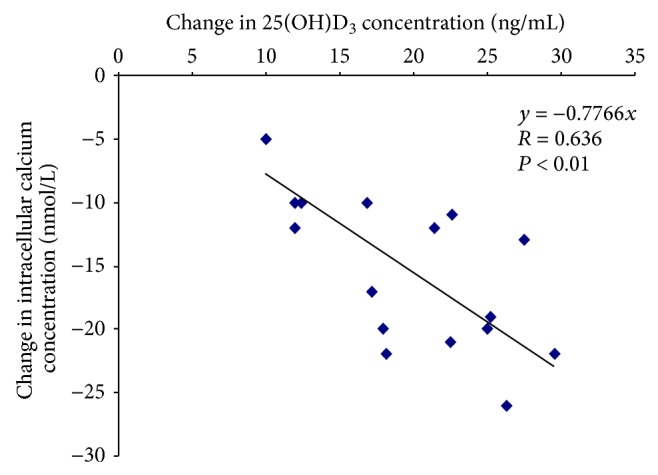
Relationships between absolute changes from baseline in [Ca^2+^]_*i*_ and 25(OH)D_3_ concentrations after a 6-month vitamin D_3_ supplementation (*P* < 0.01, *n* = 16).

Effect of cholecalciferol treatment was evaluated in 16 CKD patients (mean age 55 ± 4, range 32–80 years, 9 males and 7 females).  Table 1 shows laboratory data of CKD patients at baseline and after the vitamin D_3_ supplementation. Initial 25(OH)D_3_ concentrations were low (18 ± 8 ng/mL) and significantly increased after the vitamin D_3_ treatment (36 ± 9 ng/mL). Eleven patients reached the recommended 25(OH)D_3_ level above 30 ng/mL. The [Ca^2+^]_*i*_ in PBMCs was increased in CKD patients (120 ± 6 nmol/L). After the 6-month vitamin D_3_ supplementation, [Ca^2+^]_*i*_ significantly decreased (105 ± 3 nmol/L, *P* < 0.001, *n* = 16) to values comparable with those in healthy subjects. The absolute changes from baseline in [Ca^2+^]_*i*_ were related to the absolute changes in 25(OH)D_3_ concentrations (*P* < 0.01, *n* = 16) (Figure 1). The PTH concentrations were in reference range (43 ± 17 pg/mL) at basal conditions and vitamin D_3_ supplementation had no effect on their values. The correlation between [Ca^2+^]_*i*_ and PTH concentrations has not been found.

### 3.2. CRAC Channels

**Figure 2:**
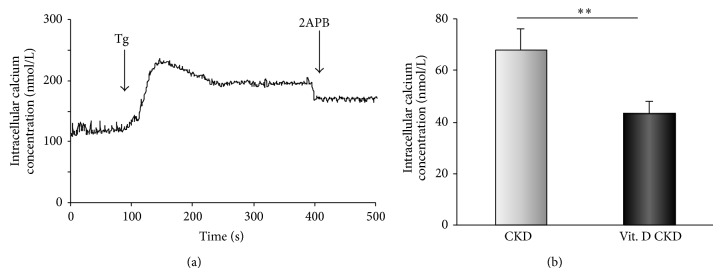
The calcium entry through CRAC channels. (a) A typical experiment where CRAC channels were activated by intracellular Ca^2+^ depletion by Tg (1 *μ*mol/L) and subsequently inhibited by 2APB (50 *μ*mol/L). The representative trace was obtained from a single experiment from PBMCs of a CKD patient. (b) The comparison of the calcium entry through CRAC channels in PBMCs of CKD patients at baseline and after a 6-month vitamin D_3_ supplementation (^**^ *P* < 0.01, *n* = 16).

CRAC channels were activated indirectly by intracellular Ca^2+^ store depletion using Tg (1 *μ*mol/L). The 2APB (50 *μ*mol/L), a reliable inhibitor of these channels, was applied during the sustained phase of Tg effect. It evoked a decrease in [Ca^2+^]_*i*_, which represented particularly the Ca^2+^ influx through CRAC channels. After the treatment with vitamin D_3_, the enhanced Ca^2+^ entry through these types of channels (68 ± 27 nmol/L) was significantly decreased (43 ± 16 nmol/L; *P* < 0.01, *n* = 16) in CKD patients (Figures 2(a) and 7(b)).

### 3.3. P2X_7_ Receptors

#### 3.3.1. P2X_7_ Channels

**Figure 3:**
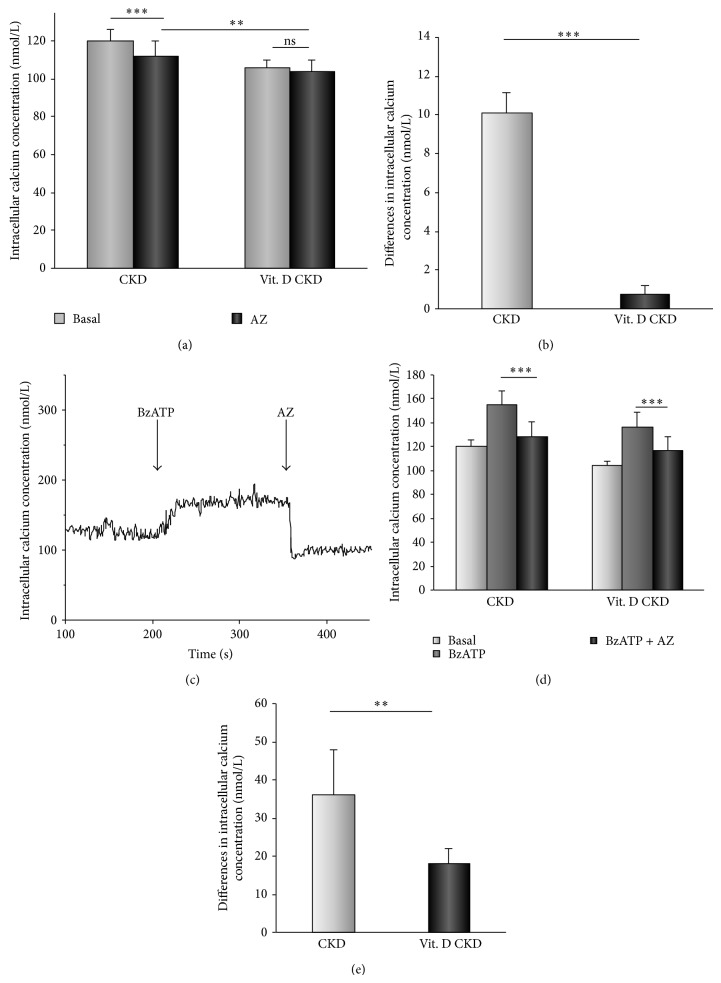
The effect of vitamin D_3_ supplementation on an inhibitory effect of AZ11645373. (a) The comparison of the AZ11645373 (50 nmol/L) effect on [Ca^2+^]_*i*_ in CKD patients at baseline and after vitamin D_3_ supplementation (^**^ *P* < 0.01, ^***^ *P* < 0.001, *n* = 16). After vitamin D_3_ supplementation, AZ11645373 (50 nmol/L) had no effect on [Ca^2+^]_*i*_. (b) Differences in [Ca^2+^]_*i*_ after an inhibition with AZ11645373 (50 nmol/L) at baseline and after vitamin D_3_ supplementation (^***^ *P* < 0.001, *n* = 16). (c) The representative trace where AZ11645373 (50 nmol/L) inhibited the [Ca^2+^]_*i*_ rise induced by BzATP (50 *μ*mol/L). (d) The comparison of the AZ11645373 (50 nmol/L) effect on BzATP- (50 *μ*mol/L) activated calcium influx before and after supplementation (^***^ *P* < 0.001, *n* = 16). (e) Differences in [Ca^2+^]_*i*_ in BzATP- (50 *μ*mol/L) activated PBMCs after the AZ11645373 (50 nmol/L) inhibition at baseline and after vitamin D_3_ supplementation (^**^ *P* < 0.01, *n* = 16).

**Figure 4:**
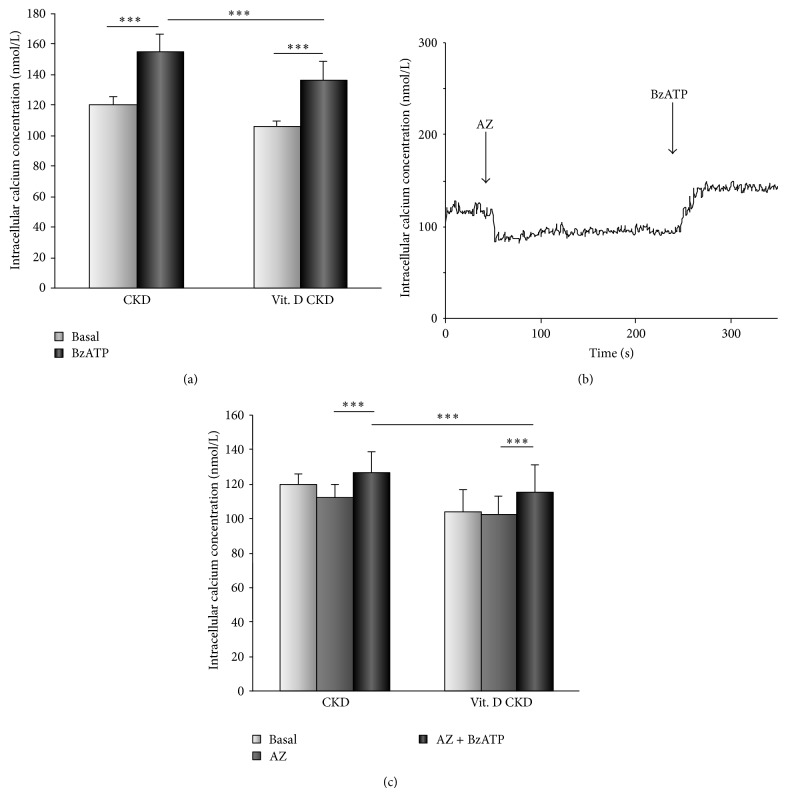
The effect of vitamin D_3_ supplementation on activation by BzATP. (a) The comparison of the BzATP (50 *μ*mol/L) effect on [Ca^2+^]_*i*_ in CKD patients at baseline and after vitamin D_3_ supplementation (^***^ *P* < 0.001, *n* = 16). (b) A typical experiment where PBMCs were pretreated with AZ11645373 (50 nmol/L) prior to the application of BzATP (50 *μ*mol/L). (c) The comparison of the BzATP (50 *μ*mol/L) effect on AZ11645373 (50 nmol/L)-inhibited calcium influx in PBMCs before and after supplementation (^***^ *P* < 0.001, *n* = 16).

The application of P2X_7_ receptors antagonist AZ11645373 (50 nmol/L) led to reduction in [Ca^2+^]_*i*_ in CKD patients from 120 ± 6 to 112 ± 8 nmol/L (*P* < 0.001, *n* = 16). On the other hand, after the vitamin D_3_ supplementation, AZ11645373 had no effect on [Ca^2+^]_*i*_ (106 ± 4 versus 104 ± 6 nmol/L; ns, *n* = 16) (Figure 3(a)). Differences in [Ca^2+^]_*i*_ after the inhibition of P2X_7_ receptors were significantly decreased after the vitamin D_3_ supplementation (*P* < 0.001, *n* = 16) (Figure 3(b)). In BzATP stimulated cells, AZ11645373 (50 nmol/L) decreased the calcium influx before and also after the vitamin D_3_ supplementation (Figures 3(c) and 3(d)), but the effect of an inhibitor was attenuated (*P* < 0.01, *n* = 16) (Figure 3(e)). The 6-month vitamin D_3_ supplementation had an inhibitory effect on function of P2X_7_ channels and thereby decreased the Ca^2+^ entry. The agonist of purinergic P2X_7_ receptors, BzATP (50 *μ*mol/L), caused a sustained increase in [Ca^2+^]_*i*_ in CKD patients at baseline 120 ± 6 to 155 ± 12 nmol/L (*P* < 0.001, *n* = 16) and also after the vitamin D_3_ supplementation 106 ± 4 to 136 ± 13 nmol/L (*P* < 0.001, *n* = 16). However, the P2X_7_ receptors activation after the vitamin D_3_ supplementation did not reach the values of [Ca^2+^]_*i*_ before supplementation (Figure 4(a)). Furthermore, the effect of BzATP (50 *μ*mol/L) on AZ11645373-inhibited calcium influx in PBMCs was evaluated. Under these conditions, a rising calcium influx through P2X_7_ channels was found at baseline and after supplementation (Figures 4(b) and 4(c)). All these results demonstrate the inhibitory effect of vitamin D_3_ supplementation on calcium entry through P2X_7_ channels.

#### 3.3.2. P2X_7_ Pores

**Figure 5:**
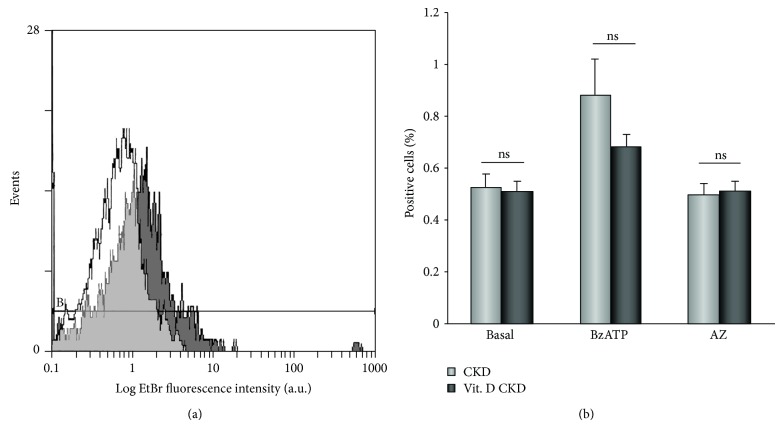
The effect of vitamin D_3_ supplementation on permeability of P2X_7_ pores. (a) Representative flow cytometry histograms of ethidium bromide entry into PBMCs of a CKD patient at basal conditions (white peak) and after a stimulation by BzATP (50 *μ*mol/L) (grey peak). (b) The comparison of the ethidium bromide permeability under the basal conditions, in BzATP- (50 *μ*mol/L) stimulated and AZ11645373- (50 nmol/L) inhibited PBMCs before and after vitamin D_3_ supplementation.

The uptake of ethidium bromide into PBMCs was measured by flow cytometry at basal conditions and with BzATP (50 *μ*mol/L) stimulation or AZ11645373 (50 nmol/L) inhibition. The permeability of ethidium bromide through P2X_7_ pores in PBMCs of CKD patients was significantly increased in comparison with healthy volunteers [4, 5] and remained unchanged after the vitamin D_3_ supplementation. The treatment did not change the permeability of P2X_7_ pores after the application of either BzATP (50 *μ*mol/L) or AZ11645373 (50 nmol/L) (Figures 5(a) and 5(b)).

#### 3.3.3. Expression of Cell Surface P2X_7_ Receptors

**Figure 6:**
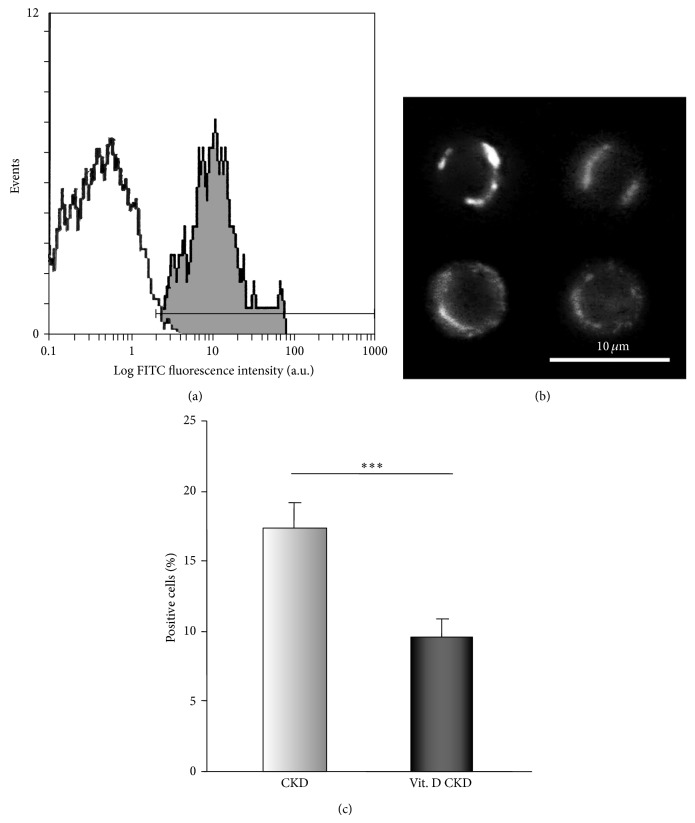
The expression of surface P2X_7_ receptors. (a) Representative flow cytometry histograms of PBMCs immunostained with primary antibody for the extracellular domain of the P2X_7_ receptor (gray peak) and an isotype-matched control (IgG2a, white peak). (b) Immunofluorescence staining of P2X_7_ receptors in plasma membrane using anti-P2X_7_ (extracellular) antibody of lymphocytes visualised by fluorescence microscopy. Images represent surface P2X_7_ receptors in four different samples. (c) Decreased expression of surface P2X_7_ receptors after vitamin D_3_ supplementation in a whole population of PBMCs (^***^ *P* < 0.001, *n* = 16).

The expression of cell surface P2X_7_ receptors was 1.5-fold greater on PBMCs from CKD patients compared to healthy donors [4]. We assessed a decreased expression of these receptors after vitamin D_3_ supplementation in the whole population of PBMCs (*P* < 0.001) (Figures 6(a), 6(b), and 6(c)).

### 3.4. Plasma Membrane Ca^2+^-ATPases

**Figure 7:**
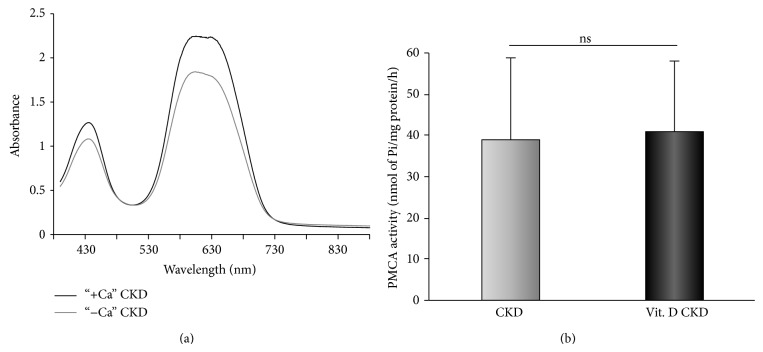
The assessment of PMCA activity. (a) The representative absorbance spectrum of a CKD patient PMCA in the medium with (black line) and without (grey line) Ca^2+^. (b) The comparison of PMCA activity in CKD patients at baseline and after a 6-month vitamin D_3_ treatment.

The PMCA activity of RBCs membranes is decreased by 25% in patients with early stages of CKD when compared to healthy subjects [4]. Vitamin D_3_ supplementation did not increase the PMCA activity (Figures 7(a) and 7(b)). The concentrations of total plasma membrane proteins were significantly enhanced (7.7 ± 1.6 versus 11.8 ± 2.7 mg/mL, *P* < 0.001) which may indicate increased expression of PMCA.

## 4. Discussion

Disturbances in intracellular calcium homeostasis in patients with CKD represent a complex process which aggravates with CKD progression. The mechanisms of cell calcium influx and efflux are impaired in renal disease. Already in early stages of CKD, cytosolic calcium concentration ([Ca^2+^]_*i*_) and calcium concentration of intracellular stores are increased [6, 7]. The elevated calcium entry through CRAC channels and P2X_7_ receptors, increased expression of P2X_7_ receptors, and decreased PMCA activity contribute to this state [4]. We have previously shown that vitamin D_3_ supplementation in CKD patients led to a decline in [Ca^2+^]_*i*_ to values comparable with healthy people [6]. The aim of this study was to examine the effect of vitamin D_3_ supplementation on predominant regulation mechanisms of cell calcium homeostasis in nonexcitable cells from patients with early stages of CKD. The principal finding of the present study is that vitamin D_3_ supplementation affected the mechanisms of intracellular calcium homeostasis as follows: the Ca^2+^ entry through CRAC and P2X_7_ channels was decreased, while no effect was found on the permeability and functionality of P2X_7_ pores, and the expression of P2X_7_ receptors was reduced. Finally, the activity of PMCA was not increased after treatment.

### 4.1. Vitamin D_3_ Supplementation

All clinical studies of vitamin D supplementation in CKD patients reported a significant improvement in 25(OH)D_3_ concentrations, although several studies did not reach a mean 25(OH)D_3_ concentration in optimal range (≥30 ng/mL). In our study, vitamin D_3_ supplementation with weekly cholecalciferol dosing significantly increased 25(OH)D_3_ to the recommended levels. PTH values were in normal range and did not change after treatment, and no correlation was observed between 25(OH)D_3_ and PTH concentrations. Total serum calcium was not changed but [Ca^2+^]_*i*_ significantly decreased after supplementation. Linear regression analysis demonstrated that changes in 25(OH)D_3_ were significantly and inversely correlated with that of [Ca^2+^]_*i*_ (*R* = 0.617, *P* < 0.01). Besides our previous work [6], no studies have investigated the relationship between vitamin D status and [Ca^2+^]_*i*_ in CKD.

### 4.2. Ca^2+^ Entry through CRAC Channels

Dysregulation of Ca^2+^ homeostasis involving the endoplasmic reticulum (ER) and store-operated calcium channels has been manifested in patients with neurodegenerative disorders, immunodeficiency, acute pancreatitis, polycystic kidney disease, and cardiac hypertrophy [9, 26–29]. In nonexcitable cells, CRAC channels are the main pathway of Ca^2+^ entry. These channels are composed of two proteins Orai1 and STIM1 (stromal interaction molecule). Orai1 protein is located in the plasma membrane and forms the channel pore. It is activated by STIM1 located in the membrane of ER. STIM1 has a dual function of sensing the Ca^2+^ concentration in ER and activating CRAC channels. A decrease in the ER Ca^2+^ concentration induces STIM1 translocation close to the plasma membrane where it binds to and activates the Orai1 channel. Alterations in STIM1/Orai1 system may contribute to several pathophysiological conditions including cardiovascular [30, 31] and pulmonary diseases [32], hypertension [33], immunodeficiency, and autoimmune and lymphoproliferative diseases [8, 34, 35]. In our previous study, we have demonstrated that the function of CRAC channels is altered in PBMCs of patients in early stages of CKD [6]. It is not known what changes in the STIM1/Orai1 system develop over the course of CKD. In the current study, a 6-month vitamin D_3_ supplementation significantly reduced the increased Ca^2+^ entry through CRAC channels which contributed to the decrease in [Ca^2+^]_*i*_. In our previous study, a 12-month vitamin D_3_ supplementation decreased the Ca^2+^ entry through CRAC channels insignificantly, which could be due to less rigorous patient selection (patients with diabetes mellitus and polycystic kidney disease were included). To our best knowledge, the effects either 25(OH)D_3_ or 1,25(OH)_2_D_3_ on CRAC channels were not studied in CKD.

### 4.3. Ca^2+^ Entry via P2X_7_ Channels

The most recent advances provide compelling evidence for P2X receptors playing a key role in regulating physiological and pathophysiological processes in the kidney [36]. In our study, P2X_7_ receptors were involved in the disrupted calcium homeostasis in PBMCs of CKD patients. We have shown that the Ca^2+^ entry via P2X_7_ channels and pores was increased and also the permeability of P2X_7_ pores was higher. In addition, the function of P2X_7_ channels and pores was altered [5]. It is known that 1,25(OH)_2_D_3_, an active metabolite of vitamin D, prevents Ca^2+^ increase through P2X_7_ channels and reduces the plasma membrane permeability through P2X_7_ pores in human PBMCs of healthy subjects [17]. 1,25(OH)_2_D_3_ can also up- or downregulate the expression of several genes in many cell types. To our knowledge, the effect of 1,25(OH)_2_D_3_ on P2X_7_ receptor expression has not been studied. The data of this study disclosed that vitamin D_3_ supplementation reduced Ca^2+^ influx through P2X_7_ channels and affected their functionality. Moreover, the supplementation had no effect on permeability of P2X_7_ pores, and the differences in responses to stimulation or inhibition were not found. Different effects of vitamin D_3_ on P2X_7_ channels and pores in CKD patients may have been made due to different sensitivity to these channels and pores. It is known that various agonists and antagonists of P2X_7_ receptors may have a distinct effect on the function of P2X_7_ channels and/or pores [37]. Not only the function but also the expression may be altered in some pathological conditions [38–41]. In our recent study, we found a 1.5-fold increase in the expression of surface P2X_7_ receptors on PBMCs from CKD patients compared to healthy subjects [4]. In the current study, the flow cytometric measurement revealed that vitamin D_3_ decreased the expression of P2X_7_ receptors by 45%.

### 4.4. Activity of PMCA

The PMCA is critical for the maintenance of resting [Ca^2+^]_*i*_ in nonexcitable cells and may be the last gatekeeper for the control of low [Ca^2+^]_*i*_. We have observed a decreased PMCA activity in early CKD patients [4]. This finding is consistent with other studies and points out that the PMCA activity decreases with kidney disease progression [42]. The decline in PMCA activity may be caused by numerous factors such as calmodulin deficiency, an activity of other endogenous protein regulators, and inhibition of mitochondrial or glycolytic metabolism [43]. The Ca^2+^ influx through CRAC channels is also an important regulator of PMCA activity, and an altered communication between them may be another cause of PMCA malfunction [44]. 1,25(OH)_2_D_3_ is known to increase PMCA expression and activity in Ca^2+^ transporting tissues such as the intestine, as well as in osteoblasts and Madin-Darby bovine kidney epithelial cells [45–47]. Several experimental studies have observed direct and/or indirect effects of 1,25(OH)_2_D_3_ and/or 24,25(OH)_2_D_3_ on activity and expression of PMCA in different cells, but the effects of 25(OH)D_3_ itself have not been studied. The studies targeting at the effects of native vitamin D_3_ supplementation on PMCA activity in patients with early stages of CKD are also missing. In the present study, we did not find changes in activity of PMCA after treatment; however, the concentration of plasma membrane proteins was increased by 47%. Therefore, we cannot preclude the possibility of increased expression of PMCA.

In conclusion, we have shown that vitamin D_3_ supplementation reduces elevated [Ca^2+^]_*i*_ via CRAC and P2X_7_ channels and decreases the expression of cell surface P2X_7_ receptors in early CKD. We did not find the effect on P2X_7_ pores and PMCA activity. Thus, vitamin D_3_ supplementation had a beneficial effect on disturbed cell calcium homeostasis in early CKD.

## Figures and Tables

**Table 1:** Main laboratory variables at baseline and after the cholecalciferol treatment.

Parameters	Baseline	6 months

Total serum calcium (mmol/L)	2.28 ± 0.08	2.34 ± 0.08
[Ca^2+^]_*i*_ (nmol/L)	120 ± 6	105 ± 3^***^
iPTH (pg/mL)	43 ± 17	43 ± 20
25(OH)D_3_ (ng/mL)	18 ± 2	36 ± 9^***^
Serum creatinine (*μ*mol/L)	102 ± 21	101 ± 28
eGFR (mL/min)	64.8 ± 5.4	63 ± 4.2

Values are expressed as mean ± SD, ^***^ *P* < 0.001,  *n*  =  16 for comparison with baseline.
